# Cell plasticity in cancer cell populations

**DOI:** 10.12688/f1000research.24803.1

**Published:** 2020-06-22

**Authors:** Shensi Shen, Jean Clairambault

**Affiliations:** 1 Inserm U981, Institut Gustave Roussy, Université Paris-Saclay, Villejuif, France; 2 Sorbonne Université, CNRS, Université de Paris, Laboratoire Jacques­Louis Lions (LJLL), & Inria Mamba team, Paris, France

**Keywords:** Plasticity, Cancer, Modelling

## Abstract

In this review, we propose a recension of biological observations on plasticity in cancer cell populations and discuss theoretical considerations about their mechanisms.

## Introduction

Phenotype switching – or, more generally, continuous phenotype-determined cell plasticity – is an essential process originally observed during development but now is also recognised as an important phenomenon upon injury and disease. In the context of anti-cancer therapies, cell plasticity enables tumour cells to change to a cell phenotypic identity that may be dependent (or not) on the drug target but without additional secondary genetic mutations. Indeed, the discovery of oncogenic-driven mutations favoured the development of diverse targeted therapies and showed unprecedented clinical response. Unfortunately, responses in general are incomplete and transient as resistances develop upon continuous treatment exposure. Along with well-known genetic alterations, cell plasticity has recently emerged as an unavoidable contributor to therapy evasion. Studying the underlying biological mechanisms of cell plasticity should not only enable a better understanding of cancer cell phenotypic and functional heterogeneity but also present novel opportunities to prevent the emergence of drug-refractory resistance. In this mini-review of recent research (focused on the years 2017–2020), we discuss adaptive behavioural phenomena in assumed genetically homogeneous cancer cell populations.

## 1. Challenging facts and possible mechanisms

### 1.1. Epithelial-to-mesenchymal transition in cancer

**Figure 1.**
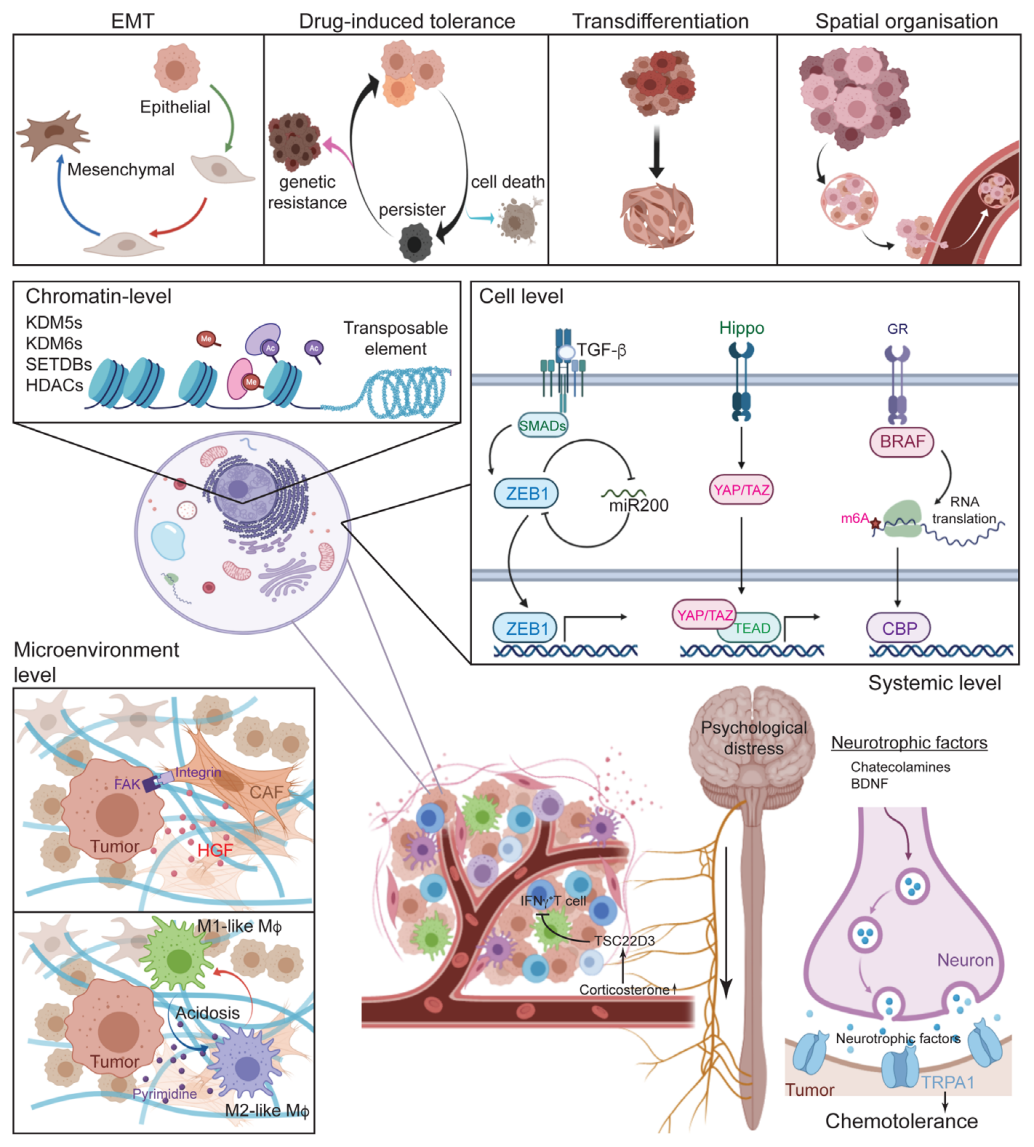
Summary of cell plasticity models. ( **A**) Four scenarios of cell plasticity represent the current models and challenges. ( **B–E**) Potential mechanisms of cell plasticity on different levels. (B) On the chromatin level, epigenetic regulators and DNA elements are shown to be involved in tumour cell tolerance to chemotherapy. (C) On the cellular level, intracellular signalling pathways sensing diverse environmental cues reprogram the transcriptional landscape, leading to adaptation to drug exposures. (D) On the microenvironmental level, inter-cellular communications build up a ‘safe haven’ by direct cell-to-cell interactions and by ‘quorum sensing’ mechanisms. (E) Psychological distress remotely controls tumour cell adaptation by secreting systemic neurotrophic factors. Ac, ; BDNF, Brain-derived neurotrophic factor; BRAF, Proto-Oncogene B-Raf; CAF, Cancer-associated fibroblast; CBP, CREB Binding Protein; EMT, Epithelial-to-Mesenchymal transition; FAK, Focal Adhesion Kinase; GR, Growth factor; HDAC, Histone deacetylases; HGF, Hepatocyte Growth Factor; IFNγ, Interferon gamma; KDM, Lysine demethylases; Me, Methylation; SETDB, SET Domain Bifurcated Histone Lysine Methyltransferase; SMAD, Mothers Against Decapentaplegic Homolog; TEAD, Transcriptional Enhancer Factor TEF; TGF-β, Transforming growth factor beta; TRPA1, Transient Receptor Potential Cation Channel Subfamily A Member 1; TSC22D3, Glucocorticoid-Induced Leucine Zipper Protein; YAP/TAZ, Yes Associated Protein/ Transcriptional Coactivator With PDZ-Binding Motif; ZEB1, Zinc Finger E-Box Binding Homeobox 1.

In various cancers, tumour cells have been shown to hijack developmental processes to adapt to environmental stresses. One of the best described examples of phenotypic switching depends on the process of epithelial-to-mesenchymal transition (EMT). It consists of both morphological and molecular changes by loss of apical–basal polarity and epithelial cell junctions accompanied by down-regulation of E-cadherin expression and acquisition of mesenchymal properties, including expression of vimentin and fibroblast-like morphology ( Figure 1A, ‘EMT’). EMT is completely reversible insofar as carcinoma cells change their phenotypes without genetic mutations. In particular, tumour cells can occupy a hybrid phenotypic state characterised by a mix of epithelial and mesenchymal features (partial EMT) and can revert to an epithelial state through the process of mesenchymal-to-epithelial transition (MET) as soon as they encounter a new environment. Indeed, coupled feedback loops of engineered network motifs by synthetic biology were shown to be able to generate intermediate hybrid EMT states ^1^. It seems that such a partial EMT phenotype is adopted by tumour cells to migrate as clusters ^2^, which we will discuss in Section 1.4.

Apart from its well-known action in metastasis, EMT has been shown to be involved in drug resistance. A recent study showed that EMT was involved in resistance to the third-generation epithelial growth factor receptor tyrosine kinase inhibitors (EGFR-TKIs) ^3^. The underlying biological mechanism by which EMT confers chemoresistance remains to be fully demonstrated. Nevertheless, transforming growth factor beta (TGFβ), a well-known EMT inducer, has been shown to be involved in EMT-related chemoresistance in *BRAF*V600E-mutated melanoma ^4^ and in a breast cancer model ^5^. These recent observations raise questions about the nature of the molecular determinants of EMT and of the associated local phenomena. Is EMT always totally reversible? To what extent is it related to the ‘plasticity’ of cancer cells?

### 1.2. Transient drug-induced tolerance

The biological mechanisms that induce cancer cell plasticity upon drug treatment remain to be fully established. Nonetheless, it seemingly involves a stepwise transition whereby tumour cells undergo a slow proliferating drug-tolerant state, called drug-tolerant persisters (DTPs), before further developing secondary mutational drug resistance ( Figure 1A, ‘Drug-induced tolerance’). Persisters were firstly described in bacteria upon antibiotic challenges ^6^. Similarly, a subpopulation of non-small cell lung cancer (NSCLC) cells engages in a reversible phenotypic change in which DTPs survive the initial onslaught of anti-cancer therapies ^7^. Similar phenomena were observed in glioblastoma and melanoma ^8– 10^.

The observations of DTPs are complemented by *in vivo* studies in a basal cell carcinoma (BCC) model ^11^. Drug removal led the BCC DTPs to switch to a proliferation state and sensitised them to vismodegib, a standard care of BCC ^12^. More recently, a single-cell study in a *BRAF*V600E-mutated melanoma showed ‘micro-heterogeneity’ amid DTPs ^13^. Indeed, that study defined four cellular states in the melanoma DTPs: starvation-like melanoma cell state (SMC), neural crest stem cell-like state (NCSC), invasive state, and pigmented state ^13^. Computational analysis suggested a transition from SMC to a bifurcation point at which DTPs can engage with diverse traits by transiting into either the NCSC or pigmented state. Peroxisome proliferator-activated receptor alpha (PPARα)-mediated fatty acid oxidation-related gene expression is likely the key transcriptional mechanism in the SMC state ^14^, whereas retinoid X receptor gamma (RXRγ) and melanocyte-inducing transcription factor (MITF) are likely responsible for the NCSC and pigmented state, respectively ^13, 15^. Taken together, these observations are compatible with the fact that a stepwise transition through a slow cycling DTP state represents a non-genetic mechanism of therapy evasion that is independent of the tumour type or treatment.

It is still not clear whether the DTPs are a pre-existing cell subpopulation or arise stochastically from dynamical fluctuation. A recent finding may favour the latter concept; Shaffer *et al.* showed that a rare population of melanoma cells transiently displayed high expression of tolerance-related genes, such as *AXL*, prior to drug exposure and is resistant to anti-BRAF treatment ^16^. This population of cells could give rise to drug-sensitive cells in a stochastic manner ^16^. These observations are reminiscent of gene expression noise due to randomness in transcription and translation. Fundamentally, the noisy expression of a gene originates from the discrete and inherently random biochemical reactions of low numbers of modules involved in the production of mRNAs and proteins, thus leading to non-genetic cell-to-cell variations. Cells rely on some combinations of variability in gene expression that could be beneficial in times of stress insults ^17^. In terms of drug resistance, a recent study showed that high expression noise of a positive-feedback network favoured adaptation under high concentration of drug exposure but that a low-noise, negative-feedback network maintained resistance by acquiring mutations ^18^. Note that such non-genetic mechanisms can also be represented in the non-stochastic, deterministic modelling framework of phenotype-structured differential equations by a diffusion term coding for non-genetic instability, standing for reversible epimutations ^19^ (see also Section 3.5).

### 1.3. Dedifferentiation and transdifferentiation

Conversion of lineage has been extensively studied in the context of development. The well-known, mainly metaphoric, Waddington’s landscape has been proposed to illustrate the fact that a progenitor cell normally rolls down within epigenetic differentiation ‘valleys’ and, owing to phenotypic bifurcations, can develop into the various finally differentiated tissue types that constitute a coherent multicellular organism (see also Section 4). In the context of cancer, dedifferentiation and transdifferentiation were observed upon therapeutic challenges, which suggests a possible plasticity in ‘cancer’s Waddington landscape’, which has metaphorically flattened valleys and lowered epigenetic barriers.

In BCC, distinct compartments of the skin epithelium are maintained by different pools of residing stem cells, including interfollicular epidermis (IFE), bulge, isthmus and sebaceous gland. Upon activation of the Hedgehog oncogenic pathway, basal cells in the IFE are reprogrammed to a bulge-like cell state and thus initiate BCC ^20^. Upon inhibition of the Hedgehog pathway, some BCC cells can transdifferentiate towards a mixed isthmus and IFE cell feature ^11^. Similar transdifferentiation was observed in prostate cancer dependent on androgen receptor (AR)-mediated signalling. Histological analysis of castration-resistant prostate cancer showed a subtype of neuroendocrine transdifferentiation in an AR-independent manner ^21– 23^. Similarly, EGFR-driven NSCLC was observed to convert towards a small cell lung cancer (SCLC) phenotype upon EGFR inhibition, thus leading to therapy evasion ^24, 25^. In rare scenarios, EGFR-driven NSCLC can also transdifferentiate into neuroendocrine histology, including large cell neuroendocrine carcinoma and small or large cell carcinoma ^26^. Interestingly, even with a mixed SCLC and NSCLC phenotype, the transdifferentiated SCLC tumour retained their original EGFR mutation, indicating that they were not *de novo* tumours. The above observations support the idea that a tiny subpopulation, diverging from the predominant cell phenotype prior to targeted therapy, may be subjected to a combination of Lamarckian plasticity and Darwinian selection upon anti-cancer therapies ( Figure 1A, ‘Transdifferentiation’).

Independent of transdifferentiation processes, simple blockade of differentiation is a characteristic of acute myeloblastic leukaemias (AMLs) ^27^. In the case of AML3, blockade occurs at the promyelocytic stage, resulting in poorly differentiated myeloblasts invading the bone marrow and later the blood of patients. Such blockade is due to the chimeric protein PML-RARα, which can be inhibited by all-trans retinoic acid (ATRA) ^28^. However, this success story in redifferentiation therapy is totally dependent on the existence of the fusion gene PML-RARα and the inhibition of the resulting chimeric protein by an adequate molecule, a situation that unfortunately has not been shown to be transposable to other cases of AML.

### 1.4. Transient spatial organisation

By functioning as an entity, collective migration provides the active and passive translocation of mobile and non-mobile cells. Such a collective cell migration may reveal worse clinical outcomes than single cells ( Figure 1A, ‘Spatial organisation’).

A recent study showed that colorectal tumour dissemination contained large clusters of epithelial cells displaying a robust outward apical pole, termed ‘tumour spheres with inverted polarity’, which propagate through the collective apical budding of colorectal cancer downstream of TGFβ signalling ^29^. This is in opposition to the traditional idea that the loss of apico-basolateral polarity is associated with the dissemination of carcinomas, suggesting that the collective tumour cell plasticity may not require the EMT program. Collective cell plasticity was also observed in breast cancer cell dissemination. However, collective cell invasion is often limited because breast cancer cells can only move through the paths structured by fibroblasts. Therefore, tumour cells may hijack the stromal fibroblasts to be the leader cells by remodelling the extracellular matrix ^30^. Similarly, during early stages of lung adenocarcinoma metastasis, these cancer cells experience an epithelial-like collective invasion and are surrounded by vimentin-positive cancer-associated fibroblasts ^31, 32^, which could also be adapted by an intermediate partial EMT program ^2^, thus further strengthening the idea that cooperation between cancer cells and normal fibroblasts can contribute to tumour collective migration and worse clinical outcome.

## 2. What is plasticity in cancer?

### 2.1. Possible definitions of plasticity

Cell plasticity is the ability of cells to change their phenotypes without genetic mutations in response to environmental cues. Pathological conditions, particularly neoplasms, have been associated with increased plasticity. For instance, Barrett’s oesophagus, a pre-malignant precursor of oesophageal adenocarcinoma, has been proposed to be such a manifestation of plasticity since it consists of the conversion of the normal squamous lining (multilayer) of the oesophagus into an intestinal-like columnar (monolayer) epithelium. Stem cells have also displayed greater plasticity when they are not within their residing tissues, leading to the proposition that the origin of cancer resides in pluripotent stem cells. However, the existence of cancer stem cells (CSCs) may not be the only way that cancer cells acquire their known plasticity. Indeed, the atavistic theory of cancer (see also Section 4.4) proposes another process through which they reach such plasticity. Epigenetic instability followed by genetic instability ^33^ in the tumour microenvironment may explain such plasticity without resorting to CSCs.

**Figure 2.**
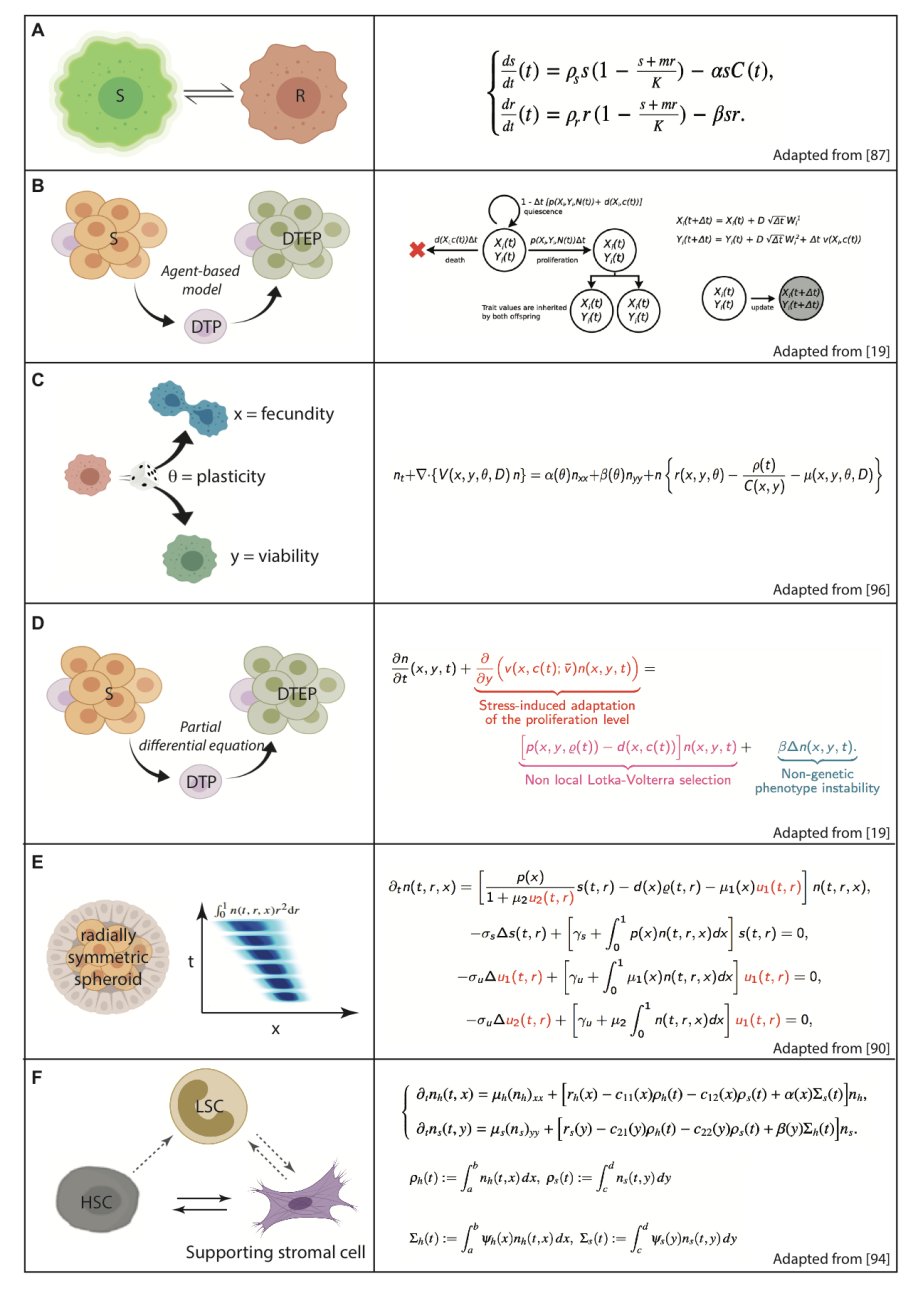
Referenced mathematical models of cell populations take cell plasticity into account by making use of ordinary differential equations (A), agent-based models (B) and phenotype-structured partial differential equations (C–F). Here, we present only the dynamics, not the initial or the boundary conditions, of the models. These models are chosen to be simple on purpose since they are all meant to provide a theoretical framework for therapeutic control and its optimisation. In particular, we do not present large systems of ordinary differential equations or large molecular networks, which nonetheless are referenced in the text. DTEP, Drug tolerant expanded persister; DTP, Drug tolerant persister; HSC, Hematopoetic stem cell; LSC, Leukemic stem cell; R, Resistant cell; S, Sensitive cell.

Open questions that arise are the following: Is plasticity a feature of cancer cell populations with binary phenotypic switch or with continuous changes (or both)? Is reversibility a common feature of cancer cell plasticity? It should be noted that from a continuous modelling point of view, the former concept allows us to deal with compartmental ordinary differential equations (ODEs) ( Figure 2A), whereas the latter implies phenotype-structured partial differential equations (PDEs) yielding completely continuous and reversible spectra of heterogeneity within the cell populations, which we will briefly develop in this review, about drug delivery optimisation in cancer cell populations. From a discrete and stochastic modelling point of view, agent-based models (ABMs) ( Figure 2B) certainly may also be used and indeed they often offer a way to justify PDE models by passing to the limit in number (N ->∞) and size (ε ->0) of cells. We firstly examine biological observations of phenotypic plasticity at different levels of multicellular organisms.

### 2.2. Chromatin level

The reversible feature of the cell plasticity that is triggered upon drug exposure, points towards a key role for transcriptional regulation at both an epigenetic level and a transcription factor level ( Figure 1B). One of the most studied epigenetic regulations in cancer cell plasticity might be histone lysine demethylase 5 (KDM5). KDM5A leads to reduced trimethylation of histone 3 lysine 4 (H3K4me3) and forms a physical interaction complex with histone deacetylase (HDAC). Sharma *et al.* ^7^ found that after treatment with an HDAC inhibitor or direct inhibition of KDM5A ^34^, DTPs restored their sensitivity to EGFR inhibition. Another KDM5 family protein, KDM5B, may bind to the promoters of cyclin-dependent kinase inhibitors (that is, p16, p21 and p27 and so on) and inhibit the transcription of these factors through demethylating the promoter-associated H3K4me3, thus leading to residual melanoma DTPs entering into a quiescent state ^35^. In addition, another histone demethylase family protein, KDM6A/B, plays a key role in glioblastoma stem cell (GSC) plasticity, in which tolerant GSCs up-regulate KDM6A/B and redistribute histone H3 lysine 27 trimethylation (H3K27me3) on repressive chromatins ^8^.

The epigenetic repression via modification of histone methylations may have considerable consequences for the activity of transposable elements. A recent study showed that H3K9me3-mediated repression of long interspersed repeat element 1 (LINE-1) was involved in the transient survival of NSCLC DTPs ^36^. In keeping with this, the authors found an accompanying up-regulation of H3K9me3-specific histone methyltransferases SETDB1 and SETDB2 in the same population, enlarging the possible role of other epigenetic factors.

Given that SETDB1 and SETDB2 are known interferon (IFN)-stimulated genes, an enrichment of IFN pathway activation was observed in NSCLC and melanoma DTPs ^37, 38^, establishing a link between methyltransferase and IFN signalling for drug-induced cell plasticity. Nevertheless, this link likely extends to adaptive resistance to immune checkpoint blockade. Benci *et al.* recently reported that prolonged IFN signalling in melanoma promotes epigenetic remodelling via transcriptional regulation of multiple T-cell inhibitory receptors, thus leading to resistance to anti-PD1 immunotherapy ^39^. An assay for transposase-accessible chromatin using sequencing (ATAC-seq) of melanoma cells showed about 50% of open chromatin regions with high STAT1 occupancy. This suggests that a consequence of this immunotherapy-induced epigenetic remodelling was to enrich IFN signalling that was also observed in NSCLC DTPs ^36^.

Adaptive mutability has also been reported in cancer DTPs. An adaptive shift towards the induction of error-prone DNA polymerases, such as Polκ and Rev1, leads to base mispairings, incorporation of aberrant DNA primer ends and thus increased mutagenesis rate ^40^. Of note, genome-wide 5-methylcytosine (5meC) hypomethylation frequently occurs in cancer genomes, leading to widespread genomic instability and de-repression of repetitive elements such as transposable elements. It is thought that hydrolytic deamination of 5meC to thymine results in T:G mismatch, which is more difficult to repair ^33, 41^. Therefore, it is plausible to hypothesise that DNA 5meC methyltransferase regulation and suppression of DNA repair gene expression cooperate to promote the development of drug resistance. However, whether a high 5meC level is associated with adaptive mutability observed in cancer DTPs still needs further exploration. Inasmuch as the regulations at the chromatin level are of an enzymatic nature with continuous activity depending on the environmental substrates, it may leave completely open the question (in Section 2.1) of plasticity as a switch-like or a continuous change of phenotypes.

### 2.3. Cell level: intracellular regulatory pathways

Chromatin level changes are generally induced by intracellular signalling cues ( Figure 1C), in which coordinated signalling networks define a specific cellular state ^42^. In the context of EMT, TGFβ induces the transcription factors Snail, Slug and ZEB1, which are each implicated in mediating the effects of TGFβ at least in part through the repression of E-cadherin. A double-negative feedback loop was observed in the TGFβ-mediated signalling response. ZEB1 expression inhibits the induction of microRNA miR-200, whereas a high level of miR-200 can inhibit the transcriptional activity of ZEB1. The TGFβ-ZEB/miR-200 double-negative feedback loop has been postulated to explain both the stability and interchangeability of epithelial versus mesenchymal phenotypes, in which mathematical modelling and experimental validation demonstrated that hysteresis control of EMT is dependent on the miR-200/ZEB1 double-negative feedback loop ^43^. Although it may not be responding directly to TGFβ, another important factor, Twist, links EMT to the ability of breast cancer cells to enter the circulation and seed metastases ^44, 45^. The mutually repressing mechanism was also seen at the network level; for instance, the BACH1 network reveals the existence of an inverse relationship between BACH1 and Raf kinase inhibitory protein (RKIP) involving both monostable and bistable transitions that potentially give rise to non-genetic variability ^46^.

The mesenchymal phenotype was also shown to be accompanied by AXL up-regulation both in NSCLC and in melanoma DTPs ^16^. Activation of AXL was associated with EMT features in erlotinib-resistant tumours and occurred either through its overexpression or via up-regulation of autocrine growth arrest-specific gene 6 (GAS6). AXL was identified as part of a gene set regulated by the transcription cofactors YAP1 and TAZ. These factors interact with TEAD1–4 transcription factors and are recruited to the *AXL* gene promoter via four TEAD-binding elements. Interestingly, a recent study showed that alternative activation of the Hippo pathway upon treatment by EGFR inhibitors in NSCLC promoted a strong epigenetic alteration driven by YAP/TEAD ^47^. Further dissection of the regulation of the YAP/TAZ/TEAD-AXL pathway will be needed at the single-cell level to examine the interactions with other signalling factors.

Apart from the well-defined EMT pathways, other intracellular signalling pathways are involved in drug-induced cell plasticity. Moparthi *et al.* showed that a FOXB2–WNT7B signalling axis induced prostate cancer transdifferentiation into neuroendocrine cell types via increased TCF/LEF-dependent transcription without activating the WNT co-receptor LRP6 or β-catenin ^48^. Intracellular signals can also be relayed through post-transcriptional modifications of mRNAs. A recent study showed that mRNA N6-methyladenosine (m6A) modification was involved in reversible resistance to BCR/ABL inhibitors in leukaemia ^49^. This is consistent with another study in melanoma DTPs ^9^. A subpopulation of m6A-associated mRNAs in their 5′ untranslated regions is up-regulated at the translational level upon BRAF inhibitor exposure ^9^. The m6A methylation is associated with mRNA stability or protein synthesis. Thus, the alternative m6A modification in DTPs may lead to a novel mRNA translation landscape. In keeping with this, Baskar *et al.* showed that mRNA translation was involved in non-heritable resistance to apoptosis induced by tumour necrosis factor alpha (TNFα)-related apoptosis-inducing ligand (TRAIL) ^50^.

### 2.4. Microenvironmental level: tumour–stroma interactions

Solid tumours are comprised of tumour cells and stromal cells, including fibroblasts, endothelial cells and infiltrated immune cells. Together with embedded extracellular matrix and vascularisation, the tumour microenvironment is involved not only in tumour growth but also in therapy-induced plasticity ( Figure 1D).

Cancer-associated fibroblasts (CAFs) are among the most well-documented cell populations in promoting therapy resistance. In melanoma, *BRAF* inhibition induces a paradoxical activation of the MAPK/ERK pathway in *BRAF* wild-type melanoma-associated fibroblasts. These cells in turn lead to elevated integrin β1-FAK-Src activation in melanoma cells undergoing treatment, generating a drug-tolerant microenvironment that provides a ‘safe haven’ for melanoma cells ^51^. Apart from direct cell-to-cell contact, CAF-secreted hepatocyte growth factor (HGF) binds to c-MET receptor on breast cancer cells, leading to alternative activation of the PI3K/AKT signalling pathway and attenuation of HER2 inhibitor sensitivity in basal-like breast cancer cells ^52, 53^. HGF/c-MET signalling is likely also involved in adaptive tolerance to cancer immunotherapy. Glodde *et al.* showed that, in the presence of HGF, neutrophils were recruited to the T cell–inflamed tumour microenvironment in response to cytotoxic immunotherapies and subsequently restrained T-cell expansion and cytotoxic T-cell effector functions ^54^.

As one of the largest stromal populations, tumour-associated macrophages (TAMs), co-evolving with tumour cells, are involved in tumour cell progression and impact on therapeutic responses. Prolonged interaction with M1-like TAMs can foster a chronic inflammation microenvironment, hence promoting cancer cell genomic instability ^55^. In this context, cancer cells acquire the ability to re-educate TAMs towards an anti-inflammatory M2-like state, which releases growth factors, pro-angiogenic molecules and immunosuppressive factors ^56^. Cancer cells and TAMs co-exist in the context of a complex, bidirectional metabolic relationship that not only is dictated by but also impinges on the tumour microenvironment ^57^. The release of CSF1, interleukin 34 (IL34) and VEGFA from tumour cells is particularly sensitive to chemotherapeutic stress, which is relevant to the metabolic symbiosis between hypoxic and normoxic cancer cells ^58^, and to the ability of lactate-producing cancer cells to repolarise TAMs towards an OXPHOS-dependent M2-like state ^59^. In melanoma, polarisation of M2-like TAMs is likely mediated by a mechanism involving a G protein–coupled receptor (GPCR) that senses microenvironmental acidosis ^60^. Importantly, the influence of cancer cells on TAMs is not unidirectional. Polarised TAMs secrete multiple cytokines with metabolic functions, including IL6, TNF, C-C motif chemokine ligand 5 (CCL5) and CCL18. Released IL6 favours tumour cell glycolysis by increasing the activity of 3-phosphoinositide-dependent protein kinase 1 (PDPK1) ^61^. It was shown that, along with cytokines, TAMs also release hypoxia-inducible factor 1 subunit-alpha (HIF1α)-stabilising long non-coding RNAs to promote the activity of HIF1α in neoplastic cells ^62^. Moreover, TAMs secrete a spectrum of pyrimidine species, such as deoxycytidine, into the microenvironment, which is taken up by tumour cells. Owing to chemical similarity, deoxycytidine taken up by tumour cells competes with gemcitabine for deoxycytidine kinase (DCK), thereby reducing its therapeutic efficacy ^63^.

### 2.5. Systemic level: neuronal factors

Among patients with cancer, emotional distress and psychiatric syndromes are prevalent during the whole period of the treatment, leading to system-level secretion of neuroendocrine hormones and neurotransmitters that could modify the tumour microenvironment and host macroenvironment ^64, 65^ ( Figure 1E). Stress sensor TRPA1, a neuronal redox-sensing Ca ^2+^-influx channel, was shown to mediate Ca ^2+^-dependent anti-apoptotic pathways and protect cancer cells against chemotherapy, suggesting that cancer cells are capable of tolerating chemotherapy-induced oxidative stress by transmitting a pain signal ^66^. This system-level control of tumour adaptive response to anti-cancer treatment has also been reported in targeted therapies. The crosstalk between β2-AR induced by catecholamines and mutant EGFR results in the expression of IL6, which can further activate STAT3 signalling to render NSCLC cells tolerant to EGFR inhibitors ^67^. Other neurotrophic factors have also been shown to be involved in the survival and stemness of glioblastoma cells. For example, the brain-derived neurotrophic factor (BDNF) secreted by differentiated glioma cells activates the neurotrophic receptor kinase 2 (NTRK2) expressed on GSCs, which promotes the activation of AKT pathways in the process of survival of GSCs ^68^. In addition to having an impact on drug treatment, psychological distress was recently shown to influence anti-tumour immunity. Yang *et al.* found that stress-elevated plasma corticosterone induced the expression of glucocorticoid-inducible factor TSC22D3 at the system level. TSC22D3 blocks type I IFN response in dendritic cells and thus abrogates IFNγ ^+^ T-cell activation in the tumour microenvironment ^69^. These results indicate that stress-induced glucocorticoid surge can subvert therapy-induced anti-cancer immunosurveillance.

## 3. Cancer cell plasticity in therapeutics

Given the emerging role of cancer cell plasticity in drug tolerance and resistance, the development of strategies targeting the underlying mechanisms of plasticity may lead to durable responses. Different strategies could be proposed by direct inhibition of cell plasticity, direct elimination of DTPs or reversing the differentiation process. Most of the strategies undergoing exploration are studied in *in vitro* models, which will necessitate a complementary *in vivo* investigation. In addition, given the toxic side effects of combination strategies, therapeutic timing and dosing issues need to be taken into serious consideration.

### 3.1. Direct inhibition of cell plasticity

Combination treatments that prevent phenotypic switching could result in a further decrease of the residual tolerant cells, thus representing an attractive strategy. At the chromatin level, given the de-repression of LINE-1 elements, the combination of erlotinib with trichostatin A or entinostat, specific inhibitors of HDAC, was shown to prevent phenotypic switching towards DTPs in subsequent studies ^36^. In glioblastoma, the combination of dasatinib with KDM6 inhibitor (GSK-J4) was shown to impair a persister cell population ^8^. Apart from epigenetic modulation, CDK7 inhibitor THZ1, which represses RNA polymerase II–mediated transcription, was shown to synergise with targeted therapies to prevent DTPs ^70^.

At the intracellular pathway level, the combination of siltuximab (an IL6 monoclonal inhibitory antibody) and ruxolitinib (a JAK/STAT inhibitor) was reported to inhibit the emergence of a neuroendocrine phenotype in human prostate cancer cells ^71^. In BCC, combination treatment of vismodegib and WNT inhibition with LGK-974 showed a reduced residual tumour cell population ^12^.

In addition to direct inhibition of tumour cell proteins, targeting components at the microenvironmental level is likely also an alternative strategy to prevent cell plasticity. Given the importance of HGF in modulating cell plasticity, combination treatment of crizotinib (c-MET inhibitor) and BRAF inhibitors suppressed CAF-mediated tolerance in melanoma ^72^. In basal-like breast tumours, treatment of PDGF-CC inhibitory antibody 6B3 can suppress the crosstalk between CAF and breast tumour cells, thus preventing transdifferentiation induced by tamoxifen or letrozole ^73^.

### 3.2. Direct elimination of drug-tolerant cells

Direct targeting DTPs in combination with chemotherapies can achieve the aim of killing sensitive and tolerant cells at the same time. Treatment with AXL antibody–drug conjugate has been shown to eliminate AXL-expressing melanoma DTPs, leading to inhibition of tumour growth in melanoma patient–derived xenografts ^74^. Shen *et al.* also showed that targeting translation initiation factor eIF4A with silvestrol could specifically eliminate melanoma DTPs ^9^. Metabolic targeting of the phospholipid glutathione peroxidase 4 (GPX4) with RSL3 was shown to induce ferroptosis of DTPs in multiple cancer models ^75– 77^. Rambow *et al.* showed that targeting RXRγ with HX531 could specifically eliminate NCSC DTPs ^13^. However, this single-cell study raises the concern that targeting a specific component may not suffice to eliminate all DTPs because it is not possible to target multi-stage DTPs simultaneously.

### 3.3. Reversal to the differentiation process

The most straightforward strategy is the ‘drug holiday’ due to the reversibility of the DTPs. Intermittent on-and-off dosing schedules have been shown to double the time of response of melanoma cells to BRAF inhibition ^78^. However, the length of the drug holidays is still difficult to determine from the clinical point of view, where patient care would be extremely complicated because of the unexpected explosive proliferation of the tumour. In addition, this simple ‘drug holiday’ strategy is highly dependent on the underlying mechanism of cell plasticity. For example, if the new cell state is maintained via hysteresis, drug holidays will not be able to reverse the phenotype for a long period of time ^43^.

In neuroendocrine transdifferentiation of prostate cancer, inhibition of EZH2 can actively reverse the lineage switch, thus restoring the sensitivity to enzalutamide treatment ^23^. Given the importance of EMT in cancer cell plasticity, actively reverting EMT by blocking TGFβ with forskolin and cholera toxin has been shown to promote MET and to sensitise these cells to anti-cancer therapies ^79^. In contrast, Ishay-Ronen *et al.* took advantage of EMT plasticity and treated mesenchymal-like breast cancer cells with rosiglitazone (PPAR inhibitor) and bone morphogenic protein 2 (BMP2) to further differentiate the tumour cells into adipocytes, thus impairing the drug tolerance ^80^. Although different strategies could be applied to control cell plasticity, insufficient evidence of clinical activity and difficulties in the determination of maximal tolerated doses and treatment schedules are still major challenges in therapeutics.

### 3.4. Simple ordinary differential equation models of epithelial-to-mesenchymal transition including transient, hybrid cell subpopulations

EMT is a paradigm of reversible cell plasticity and has been the object of many studies, not only from the experimental biology viewpoint (as mentioned earlier) but also from a systems biology viewpoint. An ODE setting has been proposed in which the model relies on gene regulatory networks involving *SNAIL*, *ZEB1*, miR-200 and miR-34 with multidirectional negative feedbacks. These networks can be controlled by external inputs on *SNAIL* expression, such as by TGFβ, nuclear factor kappa B (NFκB) and HIF1α. In a series of articles ^81– 86^, this fundamental circuit was elicited and modelled as a tristable ODE system and numerically explored, showing that between the two epithelial and mesenchymal stable states, an intermediate hybrid semistable state exists. It may be represented by a stable asymptotic branch in a bifurcation diagram, in which the bifurcation parameter may be chosen as the control input. Furthermore, in a 3D diagram (Figure 2 of ref. 86), an EMT axis has been proposed to be completed with an orthogonal differentiation axis ^86^, coding together for cell population heterogeneity in a cancer phenotype landscape whose definition remains to be made precise. Along this same line, also showing a tristable ODE system and using the same observed biological variables, another group of researchers obtained very comparable results ^87^.

### 3.5. Combined drug delivery strategies relying on heterogeneous cell population mathematical models

The first mathematical models proposed to represent drug resistance in heterogeneous cancer cell populations were compartmental ODE systems in which two distinct homogeneous subpopulations, either sensitive or resistant to anti-cancer drugs, were described by variables evolving with time under the influence of a time-scheduled drug delivery. Since then it has become possible to design optimised drug delivery strategies by using optimal control ^88^. In the 2017 study (by Carrère ^88^), the model parameters were identified from biological experiments by co-culturing epothilone-sensitive and -resistant cells of the same lineage ( Figure 2A). The optimisation problem to be solved is then to deliver the drug according to a periodic time schedule that minimises the total cancer cell population within a given finite therapeutic time window. However, one can argue that such a simplified co-culture system study ignores the reversibility of the non-genetic resistance that may be observed in clinically relevant situations ^7, 89, 90^, which raises major challenges for mathematical modelling: (1) drug resistance in these observations is reversible, and (2) it is induced by epigenetic enzymes, and any enzymatic activity, even with switching behaviour, is continuous. These two features cannot be obtained by ODE models. On the contrary, continuous phenotype-structured PDE models ( Figures 2C–F) capable of including any phenotype (that is, space, cell size, expression of drug resistance mechanisms, and so on), describing any relevant biological heterogeneity at stake in the cell population, can be applied ^91– 98^. Furthermore, drug delivery optimisation algorithms relying on optimal control can be designed on the basis of such models, leading to clinically transposable qualitative therapeutic strategies ^93, 94^. Other models that are often used in simulations of heterogeneous plastic cell populations are ABMs (also called individual-based models ^19^; Figure 2B). Contrary to PDE models, that are deterministic, taking biological uncertainty into account by second-order terms (a Laplacian) or weighted population integrals, being nevertheless totally deterministic even in their numeric simulations, ABMs explicitly introduce stochasticity in simulations of biological processes by probabilistic laws. Note that it is possible, as mentioned earlier, to integrate by averaging and passing to the limit (in particular towards infinity in number and towards 0 in size of cells) ^98^ ABMs into PDEs, easier to analyse and control. Here, we focused on proposed models of plastic cancer cell populations (sampled on Figure 2) exposed (or to be exposed) to drugs and on drug delivery strategies and not on all possible models of plasticity in cancer cell populations. However, nothing in principle opposes the utilisation of therapeutic control strategies to the dynamics of stochastic models (ABMs) of different forms of cell plasticity or on molecular and cellular networks ^99^ representing plasticity by molecular variations in very large systems of ODEs, stochastic differential equations or Boolean relations ^100– 102^ in the framework of endogenous network theory (ENT) ^103^ and the adaptive genetic landscape ^104^. Stochastic models could be called here, as they represent a relevant framework to capture the whole picture of plasticity in cancer. In particular, stochastic and ENT models ^101, 103^, as ODE models mentioned above about tristability in EMT models (that is, existence of an intermediate and transient state with intermediate characteristics between E and M states), are amenable to show an important feature of plasticity, namely the *possible* existence of transition states of phenotypes (an open question about the nature of plasticity mentioned in Section 2.1). Such transient states, as evidenced by EMT models, are of a qualitative nature and exist in a continuous range of parameters or system-determining variables, such as TGFβ or NFκB, which hopefully enable therapeutic control. In addition, so-called hybrid models (that is, both stochastic and continuous in their formulation), in which ABMs are used to represent the phenotypic behaviour of cell populations, with ^105^ or without ^98^ spatial cell growth, along with PDEs for the possible spatial diffusion of molecular species ^105^, have been proposed. Nevertheless, large systems of equations, such as the ones proposed in ENT models ^101, 103^, are not easy to handle. Indeed, one should bear in mind that the larger the system of equations, the harder it is to analyse and theoretically control, hence the mostly deterministic and not large network-like (thus here avowed as limited) point of view adopted in this mini-review, given that we aim at proposing simple models amenable to mathematical methods of optimal control to be used in the future in therapeutics.

## 4. Emergent and non-standard viewpoints on cancer biology and possible therapeutic implications

### 4.1. Tissue organisational field theory

Two main theories, representing reductionism and organicism, aim at understanding carcinogenesis. The reductionist single mutation theory (SMT) (the dominant theory) proposes that cancer is due to the genetic mutations of a single ‘renegade’ cell that begets all subsequent cancer cells. In contrast, the organicist tissue organisational field theory (TOFT) ^106, 107^ proposes that disorganised tissues favour the emergence of neoplastic transformations. SMT and TOFT differ fundamentally from each other on the basis of aspects of the default cell state (quiescent or proliferative), reversibility (irreversible or reversible) and therapeutic strategy (killing cancer cells or exploiting tissue reversibility). Although cell plasticity is by no means central in SMT or in TOFT, it may be attached to both by a general ‘principle of variation’ ^108^.

### 4.2. The Waddington epigenetic landscape revisited

Although the epigenetic landscape of Waddington was initially conceived as metaphoric ‘stemness potential’ valleys, Huang proposed the bifurcations between valleys of differentiation corresponding to stable asymptotic branches in bistable systems of ODEs ^109^. Huang *et al.* ^110^ (2007) proposed a detailed example of the *PU.1/GATA1* system of transcription factors which determines hematopoietic cell fates between the erythroid and myelomonocytic lineages. As mentioned in Section 1.3, this viewpoint allows us to illustrate plasticity in a population of cells endowed with the same genome by the instability of trajectories, in which appear ponds of non-differentiation, named ‘cancer attractors’ in which cells proliferate in a poorly differentiated state (this is illustrated on Figures 6 and 7 of Ref. 109). In this perspective, plasticity may be seen as a disorganisation of the epigenetic landscape, in which both differentiation and proliferation are uncontrolled. Note that ‘epigenetic’ here clearly means related to non-genetic modifications of the genome, due to differentiation, but in a sense that is completely independent of the molecular level of modifications of the chromatin (the ‘independence of senses’ refers to the opposition between the metaphoric representation of differentiations in the Waddington epigenetic landscape and the molecular determination of differentiations at the chromatin level). This point of view on plasticity in cancer is described by Pisco *et al.* ^89, 90^ and Huang *et al.* ^109, 110^.

### 4.3. The atavistic theory of cancer

In 2011, physicists Paul Davies and Charles Lineweaver and oncologist Mark Vincent independently advocated the idea that ‘cancer is a de-repression of a default survival program common to all cells’ ^111^, which was expressed by Davies and Lineweaver as the ‘atavistic theory of cancer’ ^112, 113^. According to this theory, cancer is a disease of the evolution of multicellular organisms in which a localised collection of cells organises itself and proliferates for its own benefit. This hypothesis has been assessed from phylostratigraphic analyses of the genomes of different species that allowed investigators to establish links between the genes that are essential to multicellularity and those that are altered in cancer ^114, 115^ and from more recent studies that elicit disrupted relationships between genes of multicellularity and genes that are disrupted in cancer ^116– 119^. Of course, this emerging field of research presupposes that cancer is an evolutionary disease of multicellular organisms; ‘evolutionary’ here means related to the Darwinian evolution of living species. In this respect, plasticity is clearly related to loss of control of the differentiations that make a multicellular organism coherent and functional, and any disruption in this control may lead to cancer without necessarily resorting to so-called CSCs. Along the same lines, it has been proposed that cancer cells are potentially resistant to letal drug insults by using bet-hedging strategies to diversify epigenetic programs. This point of view is developed in three review articles ^120– 122^. It is completely compatible with the representation of Waddington epigenetic landscape within which plasticity may be seen as a ‘flattening’ of the landscape by insufficient immune control on differentiations. More generally, independently of this metaphoric representation, we advocate that loss of control on differentiations, which is most likely related to defective control by the immune system (a view compatible with the ENT ^104^ framework), *is* actually the plasticity of cancer.

### 4.4. Philosophy of cancer: cancer and the definition of an organism

The non-standard points of view mentioned above all lead to fundamental questions: What is an organism? What does cancer have to do with the underlying conception of an organism? These questions call for the intervention of philosophy of biology and in this case for the emergent philosophy of cancer.

To make things clear, cancer being a disease of multicellular organisms (a cancerous bacterium simply does not make sense), the question of ‘what exactly is an organism?’ indeed arises quite naturally and this has been the case since Aristotle ^123^. It has been proposed that this notion may be defined in terms of the opposition between self and non-self, naturally introducing the immune system in this process. However, Thomas Pradeu ^124, 125^, presenting this question in most of its biological aspects, refutes this opposition as a working definition of an organism, proposing that the outskirts of a territory where there is immune response, that is, harsh immune interactions, are the limits of an organism. Whereas immune tolerance in the continuity of mild interactions between its components, on the contrary, defines what constitutes the inland territory of an organism. This quasi-phenomenological view of a multicellular organism, which ignores the genetic/epigenetic design (metaphorically illustrated by the Waddington epigenetic landscape relative to a single genome), has the merit of proposing an essential role for the immune system in answer to the question ‘What exactly is an organism?’ It also clearly presents cancer as a ‘deunification of an individual’ ^125^. Nevertheless, we contend that this role of the immune system should not be thought of as only a functional one that is limited to the defence against pathogens. We propose that the epigenetic processes that control differentiations and are impaired in cancer – precisely whose alteration is cancer plasticity – are defined according to an ‘immune code’, which might be related to the major histocompatibility complex of vertebrates or to its forerunners in evolution. We thus suggest that the major role of the immune system, at least as important as to fight against pathogens, is to keep the integrity of the organism – hence defining it – by controlling differentiations. This establishes a link (common to all cancers) between the plasticity of cancer cell populations and an impairment of the immune system. It is consistent with views expressed by Guler *et al.* (2017) ^36^, who showed that DTPs escaped the IFN pathway, normally induced by repeat sequences, and with other views, expressed much earlier ^126, 127^, about ‘Urmetazoa’ that need a working immune system for their stability.

The importance of CSCs ^128^ has been put forth as the main determinant of cancer. It is currently the leading view in cancer biology, according to SMT (see Section 4.1), that cancer may be explained by a single renegade cell, the behaviour of which must be understood to unravel the complexity of cancer mechanisms. Unfortunately, the study of CSCs is often pursued per se and widely neglects that cancer is a disease of multicellular organisms with heterogeneous plasticity in cancer cell populations (that is, disrupted control of their differentiations).

A general view of ancient and recent conceptions of cancer – nevertheless without the immunological vision that we and Thomas Pradeu advocate – may be found in Marta Bertolaso’s book ^129^. She does not choose between SMT and TOFT; rather, she proposes that the two theories are not necessarily incompatible with each other, a view that is also present in the ENT ^104^ framework. In fact, we also propose that the essential resides elsewhere, possibly through the involvement of the immune system, and that an understanding of heterogeneity and plasticity in cancer should involve genetics/epigenetics, mathematical modelling, evolutionary biology and immunology. This is still a long transdisciplinary way to go.

## 5. Concluding remarks

In this mini-review, we have tried to report striking observations and recent possible explanations of such facts, including less recent observations, that led us to explore the fields of cancer cell biology and medicine, systems biology, mathematical modelling and analysis, evolutionary biology, immunology, and philosophy of biology.
